# Implication of Gut Microbiota in Cardiovascular Diseases

**DOI:** 10.1155/2020/5394096

**Published:** 2020-09-26

**Authors:** Wenyi Zhou, Yiyu Cheng, Ping Zhu, M. I. Nasser, Xueyan Zhang, Mingyi Zhao

**Affiliations:** ^1^Department of Pediatrics, The Third Xiangya Hospital, Central South University, Changsha, Hunan 410013, China; ^2^Guangdong Cardiovascular Institute, Guangdong Provincial People's Hospital, Guangdong Academy of Medical Sciences, Guangzhou, Guangdong 510100, China

## Abstract

Emerging evidence has identified the association between gut microbiota and various diseases, including cardiovascular diseases (CVDs). Altered intestinal flora composition has been described in detail in CVDs, such as hypertension, atherosclerosis, myocardial infarction, heart failure, and arrhythmia. In contrast, the importance of fermentation metabolites, such as trimethylamine N-oxide (TMAO), short-chain fatty acids (SCFAs), and secondary bile acid (BA), has also been implicated in CVD development, prevention, treatment, and prognosis. The potential mechanisms are conventionally thought to involve immune regulation, host energy metabolism, and oxidative stress. However, numerous types of programmed cell death, including apoptosis, autophagy, pyroptosis, ferroptosis, and clockophagy, also serve as a key link in microbiome-host cross talk. In this review, we introduced and summarized the results from recent studies dealing with the relationship between gut microbiota and cardiac disorders, highlighting the role of programmed cell death. We hope to shed light on microbiota-targeted therapeutic strategies in CVD management.

## 1. Introduction

Cardiovascular disease (CVD), with its rising prevalence rate and mortality, entails both health threats and economic burdens to our society. As a chronic progressive condition, the development of CVDs often begins with risk factors like obesity, type 2 diabetes, and hypertension, most of which would irreversibly damage vascular structure and eventually lead to detrimental clinical outcomes like arterial thrombosis and ischemic stroke. While heredity can only be blamed for less than 20% occurrence of CVDs, dietary and nutritional statuses are two stimuli with more profound and lasting impacts [1]. Therefore, increasing evidence has suggested a close relationship between gut microbiota and CVD development [2].

The gut microbiota refers to trillions of commensal microorganisms located in the intestine in a certain proportion, whose balance is easily disturbed by food intake, lifestyle, and environment [3]. Considered a complex organ, the microbial community is required in the committed step through which food would be converted into small compounds and metabolites, thus modulating intestine structure, gut barrier integrity, inflammatory status, and host metabolism both directly and indirectly [4]. Since Hippocrates claimed that “all diseases begin in the gut” centuries ago, a great body of research has demonstrated the interplay between intestinal microbiota and diseases, including colorectal cancer [5], cerebral ischemia-reperfusion injury [6], liver fibrosis [7], and CVDs [8]. The gut microbiota accounts for 0.2–2.0 kg of the weight of an adult and approximately 50% of the dry weight of adult feces. The enormous genome of microbial genes and their functions are described as the microbiome, which outnumbers the human genome tremendously [3, 9]. Although the characteristics of the gut community may be inherited in early life, the composition could also be altered by external conditions [10, 11]. Appropriate gut microbiota structure and metabolite functions are essential in homeostasis maintenance, whereas gut dysbiosis contributes to atherosclerosis, hypertension, heart failure, arrhythmia, cardiac tumours, and others [12]. However, its underlying mechanisms are multifactorial and yet to be determined.

In this report, we introduce the role of gut microbiota in CVDs and summarize possible mechanisms, which may provide a theoretical basis and shed light on novel therapeutic strategies in the prevention and treatment of CVDs.

## 2. Mechanisms Underlying the Interaction between Gut Microbiota and the Host

The community of gut microbiota consists mostly of bacteria, fungi, and viruses in which the primary component is bacteria. There are 5 major families in the intestinal flora: *Bacteroidetes*, *Firmicutes*, *Actinobacteria*, *Proteobacteria*, and *Verrucomicrobia [*13*]*. Although the variety of species is abundant, the architecture of gut microbiota is comparatively fixed in different sites. However, the differences in gut microorganism quantities between locations are significant, with the ascending colon containing the largest number [13]. Under physiological conditions, more than 90% of the bacteria comprise *Bacteroidetes* and *Firmicutes*, while an elevated *Firmicutes/Bacteroidetes* (F/B) proportion is associated with CVDs [14]. Koliada et al. found that with the body mass index (BMI) in Ukraine adult population increasing, their F/B ratio raised likewise after removing other confounders such as age or smoking [15]. Subsequently, evaluation of children's gut microbiota composition and BMI had confirmed F/B ratio as a key risk indicator for childhood obesity [16]. Additionally, the F/B ratio is related to low-grade inflammation leading diabetes mellitus [17]. These diseases serve as both risk factors and stimulatives for CVDs. In addition to intestinal integrity maintenance, gut metabolites serve as essential messengers in the communication between gut microbiota and the host. Here, we review the mechanisms underlying the interaction between gut microbiota and the host, especially in CVDs.

### 2.1. Immunoregulation

Generated by fiber fermentation in the colon, short-chain fatty acids (SCFAs) include three major products, namely, acetate, propionate, and butyrate, all of which contain less than six carbons [18]. Apart from being nutrients and energy sources for intestinal epithelial cells, these small-molecule metabolites could enter the blood circulation, participate in immune regulation and inflammation modulation either by binding to G protein-coupled receptors (GPCRs) or by inhibiting histone deacetylases (HDACs) [18], and thereby influence gut homeostasis and host diseases. Laurence et al. found that SCFAs induce NLRP3 inflammasome activation and subsequent abundant IL-18 secretion in a GPR43- and GPR109A-dependent manner, thus eliciting favourable effects on intestinal integrity maintenance [19]. Of note, GPR43 and GPR109A are two receptors that are expressed on intestinal epithelial cells and some immune cells, where GPR43 mainly binds to acetate and propionate, while GPR109A is specifically activated by butyrate [20]. Studies have demonstrated that SCFAs beneficially upregulate not only the proliferation and differentiation of regulatory T cells (Tregs) but also the anti-inflammatory IL-10 secreted from Foxp3+ Tregs, which are mediated through GPR43 (also known as *Ffar2*) activation and HDAC inhibition [21]. Additionally, butyrate was shown to suppress proinflammatory factors, including IL-6, IL-12, and NO, from intestinal macrophages by HDAC inhibition [18]. Likewise, Bartolomaeus et al. recently proved that the anti-inflammatory role of SCFAs such as propionate significantly reduced the number of effector memory T cells and T helper 17 cells, thus mitigating cardiovascular damage [22]. However, the proinflammatory functions mediated by GPR41 (also known as *Ffar3*) and GPR43 were reported elsewhere [23], indicating that SCFA-induced immunoregulatory effects are dependent on the distinct cell types.

Additionally, trimethylamine N-oxide (TMAO) is generally investigated as a risk indicator for cardiovascular diseases, diabetes mellitus, nonalcoholic fatty liver disease, and other metabolic events [24–26]. As the end-product of dietary choline and L-carnitine, TMAO is converted from trimethylamine (TMA) in the liver by flavin-containing monooxygenases (FMOs), especially FMO3 [24]. However, how exactly TMAO functions to regulate homeostasis is seldom discussed. According to Sun et al., TMAO induces inflammation by activating the ROS-TXNIP-NLRP3 inflammasome, thereby contributing to endothelial dysfunction in human umbilical vein endothelial cells [27]. Similarly, Yue et al. showed that TMAO promotes the release of the inflammatory cytokines IL-1*β* and IL-18 via activation of the NLRP3 inflammasome from foetal human colon cells in a time- and dose-dependent manner [28]. Moreover, injection of TMAO was shown to significantly increase inflammatory markers, including cyclooxygenase 2, IL-6, E-selectin, and ICAM1, through the MAPK and NF-*κ*B signalling pathways, which then recruit leukocytes and induce vascular inflammation [29]. In these fine experiments in which treatments against TMAO were adopted, inflammatory damage was prevented. Taken together, the proinflammatory role of TMAO is established.

Plasma cholesterol, the key cellular membranes constituent and precursor of steroid hormones, vitamin D, and bile acids, is positively correlative with cardiovascular diseases. There are two main sources of cholesterol, with one-third being exogenous from daily dietary and the other two-third synthesized inside the body [30]. Confirmed with various models, microbial regulation is believed to be critically involved in cholesterol balance modulation [31]. To begin with, gut microbiome is reported to convert cholesterol into poorly absorbed coprostanol, reducing the risk of cardiovascular diseases [30, 32]. Further elucidation reveals that the presence of intestinal sterol metabolism A genes is responsible for such metabolism mediation [32]. Another key aspect the gut microbiota enrolled is bile acids metabolism. Bile acids deconjugation yields free bile acids as well as free glycine or taurine residues, which requires the participation of bile salt hydrolase enzymes (BSHs) [30]. The presence of BSHs was found within *Clostridium*, *Bifidobacterium*, *Lactobacillus*, and others. With higher degree of bile salts deconjugation, more free BAs were excreted into feces [30]. Primary bile acids refer to steroid molecules that result from the decomposition of cholesterol in the liver. Most of them are recycled back to the liver, while the rest enter the intestine, where they are converted into secondary bile acids by gut microbiota [33]. The most well-studied secondary bile acids are deoxycholic acid (DCA), lithocholic acid (LCA), and ursodeoxycholic acid (UDCA), which often function through their receptors, including G protein-coupled BA receptor 1 (TGR5), farnesoid X receptor (FXR), and vitamin D receptor (VDR) [33]. When bound to the TGR5 receptor, secondary bile acids cause the activation of macrophages and then the production of inflammatory cytokines [34]. Interestingly, researchers found that low concentrations of secondary bile acids bring anti-inflammatory effects, while high concentrations would instead cause damage. For example, Wang et al. demonstrated that low-dose DCA mitigates the inflammatory response in birds [35].

Additionally, these products from commensal microbiota would trigger innate immune signalling, thereby communicating with the host. Microbial-associated molecular patterns (MAMPs) including LPS or peptidoglycan are recognized by receptors like Toll-like receptors (TLRs), NOD-like receptors (NLRs), and others [4]. The strong connection between TLRs and atherosclerosis was confirmed in genetic mice researches. In the TLR4-/- apoE-/- mice model fed with cholesterol-rich diet, the size of aortic plaque was significantly reduced [36]. Interestingly, deficiency of TLR2 in myeloid cells had no influence in the development of atherosclerosis, suggesting the role of endothelial TLR2 in atherogenesis [37]. Furthermore, the development of arterial thrombosis was relative to NOD2, TLR2, and TLR9 signalling in platelets as well as TLR2 and TLR4 pathways in endothelial cells [4].

### 2.2. Energy Metabolism and Homeostasis

Among the numerous risk factors contributing to CVD, abnormal immune regulation and metabolic disorders represent two major elements. Metabolic syndromes such as obesity, dyslipidosis, hyperglycaemia, and insulin resistance are closely related to the occurrence and development of CVD. In recent years, the link between gut microbiota, metabolism, and CVD has gained much attention. For instance, Den and his coworkers considered SCFAs to carry metabolic benefits for those with a high-fat diet through inhibition of peroxisome proliferator-activated receptor gamma (PPAR*γ*), converting lipid synthesis to lipid oxidation [38]. Moreover, a fiber-rich diet upregulates the levels of SCFAs in the gut, which then promotes intestinal gluconeogenesis [39]. SCFAs accelerate the production of GLP-1 by binding to GPR41 and GPR43, therefore facilitating insulin secretion [39]. In contrast, TMAO aggravates triglyceride accumulation and lipogenesis in the livers of high-fat diet-fed mice [40]. Propionate was found to induce glycogenolysis and hyperglycaemia via the upregulation of glucagon and fatty acid-binding protein 4 (FABP4), thereby hindering the effects of insulin [41]. In mice with obesity, bile acid promotes GLP-1 secretion via the TGR5 pathway, thereby modulating blood sugar [42]. Notably, there is multiplicity in the associations between gut microbiota and their microbiome. For instance, TMAO could alter the bile acid profile and metabolism, thus contributing to liver steatosis and atherosclerosis [40, 43], whereas bile acid stimulates FMO3 expression via FXR, eventually resulting in TMAO production (Bennett et al., 2013). Moreover, butyrate was found to restore bile acid dysregulation and counteract hepatic inflammation [44].

**Figure 1:**
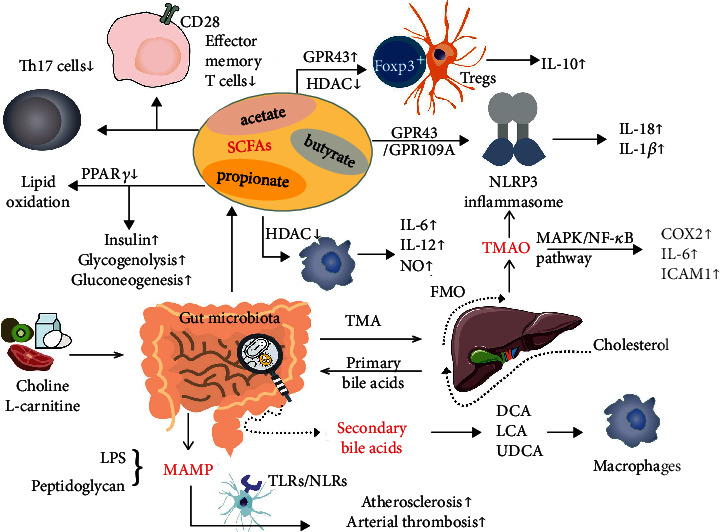
Mechanisms involved in gut microbiota-host communication. Short-chain fatty acids (SCFAs), mainly propionate, acetate, and butyrate, stimulate Fox3+ Tregs and macrophages via GPR43 activation and HDAC inhibition. Fox3+ Tregs subsequently produce the anti-inflammatory cytokine IL-10, while proinflammatory cytokines such as IL-6 and IL-12 are secreted by macrophages. Moreover, Th17 cells and effector memory T cells were downregulated by SCFAs. By suppressing PPAR*γ*, SCFAs promote lipid oxidation. Although insulin production was enhanced by SCFAs, glycogenolysis and gluconeogenesis were both observed to occur even with SCFA treatment. L-carnitine and choline consumption contribute to the release of trimethylamine (TMA), which is then converted by FMO into trimethylamine N-oxide (TMAO). Both SCFAs and TMAO activate the NLRP3 inflammasome, leading to IL-18 and IL-1*β* release. Through the MAPK/NF-*κ*B signalling pathway, TMAO increases the levels of COX2, IL-6, and ICAM1. Secondary bile acids such as deoxycholic acid (DCA), lithocholic acid (LCA), and ursodeoxycholic acid (UDCA) are produced in the intestine by gut microbiota and then participate in inflammatory modulation and blood sugar regulation.

To sum up, the gut microbiota communicates with the host through diverse manners. To begin with, SCFAs and secondary bile acids are two of the main products by gut microbiota. They play their immune-regulatory role either by directly affecting the proliferation of immune cells or by stimulating the production of cytokines. Moreover, SCFAs are involved in both lipid and sugar metabolism. Second, TMAO that primarily comes from L-carnitine and choline consumption participates in inflammatory modulation by promoting IL-18 and IL-1*β* release or activating MAPK/NF-*κ*B signalling pathway, thus upregulating the levels of COX2, IL-6, and ICAM1. Moreover, MAMPs including LPS and peptidoglycan serve as another vital contributor in the development of atherosclerosis and arterial thrombosis, mainly through TLRs and NLRs (Figure 1).

### 2.3. Programmed Cell Death

**Figure 2:**
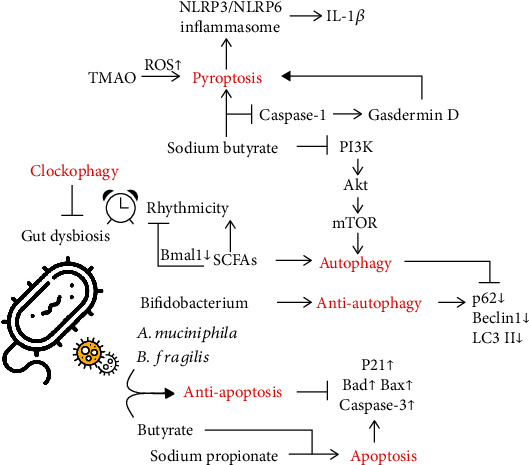
Manners of cell death induced by gut microbiota. A variety of gut flora have been demonstrated to be effective in regulating cell death. (a) Muciniphila and (b) fragilis were shown to counteract apoptosis. In contrast, sodium propionate has the ability to induce apoptosis. Interestingly, the effects of butyrate on apoptosis are controversial, manifesting elevated biomarkers such as P21, Bad, Bax, and caspase-3. In addition, SCFAs stimulate autophagy, while *Bifidobacterium* is autophagy-protective, with decreased expression of P62, Beclin1, and LC3II. Sodium butyrate promotes autophagy by inhibiting the PI3K/Akt/mTOR pathway. Additionally, it is involved in pyroptosis via regulation of the caspase-1/gasdermin D pathway. In addition, TMAO stimulates ROS activation and thus induces pyroptosis. Along with pyroptosis, the NLRP3/NLRP6 inflammasome and IL-1*β* are produced. Moreover, clockophagy can reverse gut dysbiosis. For instance, SCFAs are capable of controlling rhythmicity via clock genes such as Bmal1.

Apart from the well-known immune and inflammation modulation properties of gut microbiota, accumulating evidence has revealed its potential in the determination of diverse manners of cell death (Figure 2).

#### 2.3.1. Apoptosis

Characterized by the formation of a distinctive apoptotic body, apoptosis is one of the most widely investigated programmed cell deaths. It is often observed in myocardial infarction, heart failure, and other vascular damage. Saito et al. found that *Bacteroides fragilis* (*B. fragilis*) is able to protect HT29 cells from apoptosis resulting from Shiga toxin [45]. Butyrate promotes vascular smooth muscle cell growth via proliferation arrest as well as apoptosis inhibition [46]. Notably, there are proapoptotic effects as well. Sodium propionate was reported to induce apoptosis in H1299 and H1703 lung cancer cells, as evidenced by increased protein expression of p21, Bad, and Bax as well as apoptosis markers, including cleaved PARP and cleaved caspase 3 [47]. According to Nie et al., *Bifidobacterium* (BIF) ameliorates TNF-*α*-induced cell apoptosis in Caco-2 cells [48]. Likewise, butyrate causes apoptosis and cell cycle arrest in kidney epithelial cells [49].

#### 2.3.2. Autophagy

Nie et al. discovered that BIF ameliorates TNF-*α*-induced autophagy in Caco-2 cells by suppressing the level of p62 and inhibiting the expression of autophagy-related markers such as Beclin1 and LC3II [48]. According to their research, BIF may provide a therapeutic target aimed at the Kawasaki disease, which is highly related to acquired heart disease in children. Lannucci and his coworkers proved that SCFAs induce autophagy in hepatic cells by uncoupling protein 2 (UCP2) [50]. Accordingly, Qiao et al. demonstrated that sodium butyrate contributes to the reduction in *α*-synuclein both via the inhibition of the PI3K/Akt/mTOR autophagy pathway and enhancement of Atg5-mediated autophagy, manifested as elevated LC3II and reduced p62 expression [51].

#### 2.3.3. Pyroptosis

As a type of proinflammatory cell death, pyroptosis is characterized by swollen cells, subcellular organelle damage, and the release of cytokines, including the NLRP3 inflammasome, NLRP6, an apoptosis-associated speck-like protein containing CARD (ASC), cysteinyl-aspartate-specific proteinase 1 (caspase-1), and gasdermin D. Data have shown that sodium butyrate is capable of breaking down the gingival epithelial barrier by inducing pyroptosis [52]. Similarly, TMAO promotes vascular endothelial cell pyroptosis via ROS production, thus resulting in the development of atherosclerosis [53]. However, Gu et al. proved the antipyroptosis effects of sodium butyrate on renal glomerular endothelial cells, protecting them from damage caused by high glucose [54]. From the perspective of the mechanism, the classic caspase-1-gasdermin D pathway and NF-*κ*B/I*κ*B-*α* signalling may both be involved [54]. Moreover, Cohen et al. confirmed that *Vibrio proteolyticus* (VPRH), a Gram-negative bacterium from the gut of a wood borer, induces pyroptosis by activating the NLRP3 inflammasome and caspase-1, thereby resulting in IL-1*β* secretion, suggesting that the NLRP3 inflammasome pyroptotic pathway can benefit the host during infection [55].

#### 2.3.4. Ferroptosis

Induced by lipid reactive oxygen species accumulation, ferroptosis refers to another distinct kind of cell death mediated by mitochondria. Studies concerning whether gut microbiota are implicated in ferroptosis are rather rare. Until recently, Robert et al. proposed that supplementation of omega-3 polyunsaturated fatty acids (n-3 PUFAs) and butyrate may both facilitate mitochondrial Ca2+- and Gpx4-dependent ferroptosis [56]. Hopefully, this hypothesis may shed light on the link between gut microbiota and ferroptosis as well as accelerate related research.

#### 2.3.5. Clockophagy

The circadian rhythm, namely, clockophagy, is controlled by a complex circadian clock gene network including the ARNTL, CLOCK, CRY2, and PER2 genes [57]. The interaction between circadian rhythms and diverse gut microbiota has been well studied, where the acute sleep-wake cycle shift alters the functional profiles of gut microbes. Together, the clock-microbial communities affect host homeostasis [58]. The circadian rhythm of SCFA production was observed by Segers et al. to cause rhythmicity in intestinal movement [59]. However, such effects were abolished by the deletion of Bmal1 [59]. Besides, Marques et al. found that in hypertensive mice, a high-fiber diet changes the composition of the gut microbiota and restores gut dysbiosis, which may be partially due to increased levels of clock genes in the heart and kidney [60]. Additionally, a negative correlation between the phylum *Firmicutes* and Bmal1 as well as a positive correlation between *Bacteroidetes* and Bmal1 was observed in mice [61].

## 3. Implications of Gut Microbiota in CVDs

To concisely describe the role of gut microbiota in cardiovascular disease, the positive or negative effects of gut microbiota on CVDs are listed in Table 1.

### 3.1. Hypertension

Hypertension (HTN) has been a key link in the occurrence and development of cardiovascular diseases. Although HTN is currently beyond cure, it is preventable and controllable. According to the mosaic theory advanced by Irvin Page, HTN is induced by multiple factors, including inheritance, diet, and environment [62]. HTN also has extensive impacts on various tissues and organs, such as endothelial cells, the kidneys, and brain. Moreover, in recent years, the value of gut microbiota in HTN has been widely investigated.

In the work conducted by Li et al., fecal transplantation was performed from hypertensive individuals to germ-free mice. Along with microbiota shift, blood pressure was also elevated in those mice, indicating the contributing role of gut microbiota in hypertension [63]. It has been demonstrated that butyrate-producing bacteria and butyrate levels are relatively low in patients with HTN, indicating that imbalanced host-microbiome cross talk is relevant to systolic blood pressure [64]. Accordingly, in mice pretreated with angiotensin II, supplementation with butyrate effectively lowered blood pressure [65]. Interestingly, the same team found that gut barrier dysfunction is another contributor to HTN, as evidenced by elevated levels of zonulin, a gut epithelial tight junction protein regulator [65]. However, the same metabolite may yield contradictory biological effects through different receptors. For instance, Jennifer et al. found that propionate may upregulate blood pressure via olfactory receptor 78 (Olfr78) while exerting hypotensive effects through activation of Gpr41 [66]. In-depth knowledge reveals that vascular inflammation and endothelial dysfunction are two key processes in the development of hypertension [67]. In mice fed with Western diet, endothelial dysfunction was associated with decreased proportion of *Bifidobacterium* spp., whereas antibiotic administration helped mitigate such vascular damage [68]. As compared with germ-free mice, the conventionally raised mice pretreated with Ang II presented with a higher level of IL-4 and IL-10, indicating a vascular inflammation-prone role of enteric flora [68]. In a meta-analysis of 8 studies, a higher circulating TMAO level was positively associated with hypertension risk, which was dose-dependent [69]. Liu and coworkers identified that administration of the *Lactobacillus rhamnosus GG* strain is an effective approach to prevent exacerbation of HTN, which is in part mediated by reducing TMAO levels [70]. However, it is worth noting that the application of TMAO alone would not alter blood pressure in normotensive rats but prolonged the hypertensive-prone effects of angiotensin II [71]. More recently, a novel mechanism different from inflammation or immunity regulation has been presented. In high salt-induced hypertensive mice, elevated blood pressure is closely related to increased levels of intestinal-derived corticosterone [72].

Taken together, these results established that the gut microbiota is involved in blood pressure regulation. However, the underlying mechanisms still await further validation.

### 3.2. Atherosclerosis and Arterial Thrombosis

Initially related to dyslipidaemia, abnormal accumulation of macrophages, and massive production of inflammatory cytokines, atherosclerosis is considered a chronic inflammatory disease that underlies end-stage CVDs such as myocardial infarction or heart failure. In recent years, people have started to consider gut microbiota potent regulators during the development of atherosclerotic lesions. Koren et al. first identified bacterial DNA in atherosclerotic plaques, and the amount of DNA was associated with the infiltration of leukocytes in the plaques [73]. Moreover, the altered composition of the gut microbiome was confirmed in a metagenome-wide association study encompassing 218 individuals with atherosclerosis and 187 healthy controls. Specifically, the abundances of *Enterobacteriaceae*, *Ruminococcus gnavus*, and *Eggerthella lenta* were significantly increased in those with atherosclerosis, whereas *Roseburia intestinalis* and *Faecalibacterium* cf. *prausnitzii*, both butyrate-yielding bacteria, were reduced [74]. The above findings strongly suggest correlations between gut microbiota and atherosclerosis.

With the use of atherosclerosis-prone germ-free mice and antibiotic treatments, the role of gut microbiota in atherosclerosis development was further elucidated (Table 2). First people suggested that bacterial or viral infection is necessary for the initiation of atherosclerosis. However, such hypothesis was overturned by Samuel and his colleagues' work [75]. Apolipoprotein (apo) E-/- murine model was often adopted for atherosclerosis research given the self-driven ability of atherosclerotic plaque formation. Samuel et al. compared the atherosclerosis lesion in germ-free apoE-/- animals with those raised in conventional environment, and they found no evident difference [75]. Alternatively, with the help of antibiotics to suppress gut microflora, choline-enhanced atherosclerosis in aorta was off-set along with reduced macrophage and scavenger receptor CD36 [76]. However, given the complexity of enteric flora, the pro- or antiatherosclerosis role of gut microbiota depends. Kasahara and his colleagues demonstrated that *Roseburia intestinalis* is capable of ameliorating atherosclerosis by shaping gene expression, enhancing fatty acid metabolism, and reducing the inflammatory response [77]. However, treatment with butyrate markedly mitigates the formation of atherosclerotic plaques via the upregulation of ABCA1 and subsequent cholesterol efflux [78]. In contrast, the production of TMAO by gut microbiota yields negative effects on atherosclerosis [79].

Rupture of the atherosclerotic plaque would likely cause arterial thrombus elsewhere, resulting in detrimental consequences. For one, the LPS-TLR pathway is a m4ajor contributor in thrombosis formation. Both TLR2 and TLR4 were found expressed on endothelial cells and platelets. Activation of TLR2 and TLR4 pathway would facilitate the release of VWF and factor VIII expression, contributing to platelet-proinflammatory cell aggregation [80]. For another, gut microbiota metabolites take part in arterial thrombosis as well. Feces transplantation of TMAO-rich gut microbiota into germ-free mice would promote platelet function and arterial thrombosis [81]. Recently, another gut microbial metabolite, Phenylacetylglutamine (PAGln), was shown to induce hyperreactivity of platelet via adrenergic receptors [82].

### 3.3. Myocardial Infarction

The connection between intestinal flora and myocardial infarction (MI) has been supported by a growing body of literature. In a rat model of acute myocardial infarction (AMI), enrichment of the *Synergistetes* phylum, *Lachnospiraceae* family, *Spirochaetes* phylum, *Syntrophomonadaceae* family, and *Tissierella and Soehngenia* genera was observed compared with the sham group, which is in parallel with gut barrier impairment [83]. In patients with ST-elevation myocardial infarction (STEMI), systemic microbiome alteration was also observed. Over 12% of plasma bacteria were identified to originate from the gut after STEMI, which is partially associated with the inflammatory response [84]. Accordingly, reduced cardiac damage and decreased inflammation were noticed following the abrogation of bacterial translocation [84]. Of clinical value, plasma TMAO levels may be potential markers to predict the risks of incident cardiovascular events in patients presenting with chest pain [85]. Such potency may in part be explained by TMAO-related proinflammatory monocyte augmentation [85]. Moreover, Tang et al. demonstrated that gut microbiota-derived SCFAs would benefit cardiac repair and improve post-MI outcome though modulation of immune composition [86]. With the administration of the probiotic *Lactobacillus plantarum* 299v, the leptin level in blood was reduced, leading to enhancement of ischemic tolerance in the myocardium and alleviation of acute cardiac injury after MI [87].

### 3.4. Heart Failure

As an irreversible end-stage disease, heart failure (HF) is characterized by oedema and dyspnoea, with a five-year mortality rate of over 50% [88]. At present, a growing body of research has confirmed the “gut hypothesis of heart failure” [89, 90]. That is, decreased cardiac output in HF leads to intestinal mucosa barrier damage and dysbacteriosis, with elevated levels of pathogenic bacteria such as *Candida* [91] and reduced levels of anti-inflammatory bacteria such as *Faecalibacterium prausnitzii* [3]. Reciprocally, intestinal flora promotes HF development by participating in mucosal immunity modulation [3]. Segmented filamentous bacteria can stimulate the secretion of IL-6 and IL-23 and then promote the differentiation of Th17 cells. *Bacteroides fragilis* increases the abundance of Foxp3+ Treg cells and induces the secretion of anti-inflammatory cytokines, which have been found to reduce ventricular remodelling in MI mice [92].

Not surprisingly, metabolites of intestinal flora are also important for HF. Although studies concerning SCFAs and HF are limited, it has been proven that SCFAs are beneficial for the intestinal mucosa [3]. The depletion of SCFAs would result in intestinal barrier destruction, which then facilitates the translocation of endotoxin into blood circulation and finally leads to HF [93, 94].

However, the level of TMAO has long been recognized as a risk factor. Savi et al. found that TMAO promotes the release of calcium ions in cardiac muscle cells of healthy mice and thus alters their contractility [95, 96]. Recently, the in-depth work carried out by Jin et al. showed that TMAO confers detrimental effects on adult cardiomyocytes by inducing T-tubule network damage and Ca handling dysfunction [97]. When TMAO was administered to HF mice, Organ et al. found that mouse cardiac function deteriorated significantly, characterized by pulmonary oedema, cardiac enlargement, and decreased ejection fraction [98]. Schuett et al. proved that TMAO could enhance patient susceptibility to HF by increasing myocardial fibrosis [99]. Likewise, Wang and his team proved that 3,3-dimethyl-1-butanol (DMB) ameliorates adverse cardiac structural remodelling in overload-induced HF mice by downregulating TMAO levels [100]. Given the critical role of TMAO in HF, it may serve as a potential therapeutic target.

### 3.5. Arrhythmia

Arrhythmia, including atrial fibrillation (AF), ventricular arrhythmia (VA), and atrioventricular block, is emerging as intractable CVD that contributes to heart failure or sudden cardiac death. Up-to-date studies have shown that anticancer therapies may induce cardiotoxicities, such as corrected QT interval prolongation and arrhythmia [101]. Additionally, Vahdatpour et al. found that atrial arrhythmia can be secondary to chronic lung disease-associated pulmonary hypertension [102]. Due to its prevalence and accompanying adverse events, investigation about arrhythmia has deepened, and we are now looking at the implications between gut microbiota and arrhythmia.

Zuo et al. previously identified variable metabolic patterns as well as imbalanced gut microbiota composition in patients with AF in which *Ruminococcus*, *Streptococcus*, and *Enterococcus* significantly increased while *Faecalibacterium*, *Alistipes*, *Oscillibacter*, and *Bilophila* obviously reduced [103]. Later, they found that patients with persistent AF (psAF) shared a great proportion of common features of gut microbiota dysbiosis [104]. In their latest study, the fecal microbiota from patients with psAF and those with paroxysmal AF were investigated, verifying a similar pattern of gut microbiota, with similar ratios of *Firmicutes* to *Bacteroidetes* [105].

Svingen et al. conducted a study in thousands of patients with suspected stable angina and proposed that plasma TMAO levels are definitely related to AF [106]. It is well known that thrombi can easily take place in the left atrial appendage of patients with AF, which then leads to embolism. Gong et al. found that in patients with AF, elevated TMAO levels are related to thrombus formation, manifested as platelet hyperreactivity [107]. It has been confirmed that the cardiac autonomic nervous system (CANS) can regulate the pathophysiology of AF or VA [108]. Meng et al. first proposed that preserving dysbacteriosis or modulating metabolites such as TMAO may be a target to treat arrhythmia due to the ability of TMAO to stimulate CANS and deteriorate ischaemia-induced VA by releasing proinflammatory markers such as IL-1*β* and TNF-*α* [109]. Similarly, according to the experiment of Yu et al., gut microbes have the ability to counteract AF progression by producing TMAO and can thus activate CANS in a rapid atrial pacing-induced canine AF model [110]. Likewise, in a propionate-treated hypertensive mouse model, the susceptibility to cardiac ventricular arrhythmias was significantly reduced, indicating possible links between SCFAs and arrhythmia development [22]. Although the connection between gut microbiota and arrhythmia has been established, the precise underlying mechanisms still await further investigation (Table 2).

## 4. Microorganism-Targeted Therapies

### 4.1. Fecal Microbiota Transplantation

As an effective approach to directly introduce intestinal flora, fecal microbiota transplantation (FMT) has gained much attention. The therapeutic value of FMT in gastrointestinal diseases, neurological and psychiatric disorders, and immunology regulation has been extensively examined [22, 111, 112]. However, studies concerning its application in CVDs are limited. Although oral supplementation of resveratrol has been proven to improve glucose homeostasis by altering gut microbiota, in the work of Kim and his colleagues [113], FMT from resveratrol-fed mice to obese mice was found to yield better results than oral administration of resveratrol alone, indicating that FMT is more straightforward and direct. Moreover, Hu et al. showed that FMT could abolish the increased proportion of *Firmicutes*/*Bacteroidetes*, diminish inflammatory infiltration in cardiomyocytes, and thereby attenuate myocarditis in mice [5]. However, in a double-blind trial involving 20 patients, the composition of intestinal flora was altered in the recipients after FMT from vegetarians, whereas the vasculitis indicators presented no improvement [114]. There are also disadvantages to FMT. For instance, endotoxins are transferred along with the donor microbiome. How to weigh the pros and cons of actual practice is still an issue to be addressed. To guarantee the reliable and smooth application of FMT in clinical use, the establishment of stool banks is on its way.

### 4.2. Probiotic Administration

Among the numerous bacteria residing in the host intestine, some are beneficial. An extra boost of these bacteria would probably bring positive results, thus leading to the application of probiotics. In a meta-analysis involving 846 individuals with hypertension, mild reductions in blood pressure, body mass index (BMI), and blood glucose levels were observed after probiotic administration, supporting the beneficial role of probiotics in blood pressure control [115]. Similarly, in other studies with spontaneously hypertensive rats, the probiotics *Bifidobacterium breve* and *Lactobacillus fermentum* were found to elicit antihypertensive effects by restoring gut microbiota balance and preventing endothelial dysfunction [116], whereas long-term supplementation with kefir ameliorated high blood pressure via improvement in intestinal integrity [117]. Moreover, in apoE-/- mice fed with HFD, supplementation with *Lactobacillus rhamnosus* GR-1 markedly reduced atherosclerotic lesion size by alleviating oxidative stress and inflammation [118]. Likewise, *Lactobacillus plantarum* ZDY04 has been shown to downregulate serum TMAO levels, which is a critical factor contributing to atherosclerosis development [119].

### 4.3. Herbal Medicine

Traditional Chinese medicine (TCM), which mainly utilizes herbs and their extracts, has recently been demonstrated to treat CVDs via intestinal microbial modulation. Ou et al. reviewed and summarized the mechanisms of gut flora in TCM's theory of “stasis of intermingled phlegm and blood stasis” [120]. For example, the fact that TMAO promotes thrombosis might be one of the major causes of CVDs [121]. Anlu et al. showed that berberine originating from the Chinese herb *Coptis chinensis* has the ability to regulate the “microbiota-metabolism-immunity” axis [122]. Moreover, resveratrol derived from *Polygonum cuspidatum* was demonstrated to attenuate TMAO-induced atherosclerosis in apoE-/- mice by remodelling microbiota as well as decreasing TMAO and BA levels [123]. In addition, Ghosh et al. found that curcumin, a phytochemical component of *Curcuma longa*, attenuates atherosclerosis in LDLR-/- mice by regulating intestinal barrier function [124]. Anwar et al. showed that Trigonelline, which is purified from the seeds of *Trigonella foenum-graecum*, can inhibit the growth of *Citrobacter freundii* and subsequently decrease the production of TMAO in mice [125].

## 5. Conclusion

Evidence from a compilation of studies of animals and humans indicates that the implications of gut microbiota and their metabolites in CVDs are well established. With high-throughput technologies, verification of the intestinal flora composition and in-depth mechanistic exploration are accessible. However, the links between gut microbiota and disease development are so complex that they involve immune regulation, the inflammatory response, gut barrier integrity, metabolic homeostasis, etc. Further investigations into the specific mechanisms are needed, which then share the possibility of being transferred into clinical practice.

## Figures and Tables

**Table 1:** The exact role of different gut microbiota in CVDs.

CVDs	Atherosclerosis	Myocardial infarction	Heart failure	Arrhythmia
Species				
*Enterobacteriaceae*	Negative			
*Ruminococcus gnavus*	Negative			
*Eggerthella lenta*	Negative			
*Roseburia intestinalis*	Positive			
*Faecalibacterium* cf. *prausnitzii*	Positive			
*Synergistetes phylum*		Negative		
*Lachnospiraceae* family		Negative		
*Spirochaetes* phylum		Negative		
*Syntrophomonadaceae* family		Negative		
*Tissierella* and *Soehngenia* genera		Negative		
*Lactobacillus plantarum* 299v		Positive		
*Faecalibacterium prausnitzii*			Positive	
*Bacteroides fragilis*			Positive	
*Ruminococcus*				Negative
*Streptococcus*				Negative
*Enterococcus*				Negative
*Faecalibacterium*				Positive
*Alistipes*				Positive
*Oscillibacter*				Positive
*Bilophila*				Positive

**Table 2:** Researches of gut microbiota in CVDs.

Diseases	Sample	Observations	Mechanism	Ref.
Hypertension	HTN patients	Decreased butyrate-producing bacteria and butyrate level	SCFA-dependent	[62]
	Ang-II pretreated mice	Reduced BP after butyrate administration; increased zonulin level	SCFA-dependent; gut barrier dysfunction	[65]
	Mice	Increased BP after propionate treatment	Olfr78-dependent	[66]
	Mice	Decreased BP after propionate treatment	Gpr41-dependent	[66]
		*Lactobacillus rhamnosus* GG prevents HTN development	Reduced TMAO levels	[70]
	Mice	High salt-induced HTN	Increased intestinal-derived corticosterone	[72]
Atherosclerosis	Patients	Bacterial DNA observed in atherosclerotic plagues	/	[73]
		*Roseburia intestinalis* ameliorates atherosclerosis	Alter gene expression, induce fatty acid metabolism, and reduce inflammation response	[77]
	apoE-/- mice	Comparable atherosclerosis lesion in germ-free apoE-/- animals and their conventionally raised counterparts	/	[75]
		Choline-enhanced atherosclerosis in aorta was off-set by antibiotics	Reduced macrophage and scavenger receptor CD36	[76]
	apoE-/- mice with HFD	Butyrate mitigates atherosclerotic plaque formation	Upregulation of ABCA1 and subsequent cholesterol efflux	[78]
Myocardial infarction	AMI rat model	Increased *Synergistetes phylum*, *Lachnospiraceae family*, *Spirochaetes phylum*, *Syntrophomonadaceae* family, and *Tissierella* and *Soehngenia* genera	In parallel with gut barrier impairment	[83]
	STEMI patients	Over 12% plasma bacteria originated from the gut	Partially associated with an inflammatory response	[84]
	Patients presenting with chest pain	Predictive value of plasma TMAO levels for incident cardiovascular events	TMAO-related proinflammatory monocytes augment	[85]
	Mice	Improve cardiac repair and post-MI outcome though modulation of immune composition	Gut microbiota-derived SCFAs modulate immune composition	[86]
		*Lactobacillus plantarum* 299v improved ischemia tolerance and acute cardiac injury after MI	Reduce leptin level	[87]
Heart failure	Mice	*Bacteroides fragilis* reduces ventricular remodelling	Increased Foxp3+ Treg cells and anti-inflammatory cytokine	[92]
		Depletion of SCFAs finally leads to HF	Intestinal barrier destruction, with endotoxin translocation	[93, 94]
	Mice	TMAO alters cardiac muscle cells contractility	Promotion of calcium ions release	[95, 96]
		TMAO confers detrimental effects on adult cardiomyocytes	T-tubule network damage; Ca handling dysfunction	[97]
	Mice	Pulmonary edema, cardiac enlargement, and decreased ejection fraction	TMAO-dependent	[98]
	Patients	TMAO increases susceptibility to HF	Induction of myocardial fibrosis	[99]
	Overload-induced HF mice	DMB ameliorates adverse cardiac structural remodelling	Downregulating TMAO levels	[100]
Arrhythmia	Patients	Shared common features of gut microbiota dysbiosis	Alike ratio of *Firmicutes* and *Bacteroidetes*	[104, 105]
	Patients	Thrombus formation; platelet hyperreactivity	Elevated TMAO level	[107]
		TMAO stimulates ischemia-induced VA	Release of proinflammatory markers such as IL-1*β* and TNF-*α*	[109]
	Canine AF model	Gut microbes counteracts AF progression	TMAO production and CANS activation	[110]
	Mice	Reduced susceptibility to cardiac ventricular arrhythmias	SCFA-dependent	[22]
